# Treatment of intracranial meningioma with single-session and fractionated radiosurgery: a propensity score matching study

**DOI:** 10.1038/s41598-020-75559-8

**Published:** 2020-10-28

**Authors:** Sheng-Han Huang, Chun-Chieh Wang, Kuo-Chen Wei, Cheng-Nen Chang, Chi-Cheng Chuang, Hsien-Chih Chen, Ya-Jui Lin, Ko-Ting Chen, Ping-Ching Pai, Peng-Wei Hsu

**Affiliations:** 1 Department of Neurosurgery, Chang Gung Memorial Hospital at Linkou, No. 5, Fu-Xing Street, Kwei-shan Dist., Taoyuan, 33305 Taiwan, Republic of China; 2 Department of Radiation Oncology, Chang Gung Memorial Hospital at Linkou, Chang Gung University, Taoyuan, Taiwan, Republic of China; 3 Department of Neurosurgery, Chang Gung Memorial Hospital at Keelung, Chang Gung University, Keelung, Taiwan, Republic of China

**Keywords:** Medical research, Oncology

## Abstract

Single-session stereotactic radiosurgery (SSRS) is recognized as a safe and efficient treatment for meningioma. We aim to compare the long-term efficacy and safety of fractionated stereotactic radiotherapy (FSRT) with SSRS in the treatment of grade I meningioma. A total of 228 patients with 245 tumors treated with radiosurgery between March 2006 and June 2017were retrospectively evaluated. Of these, 147 (64.5%) patients were treated with SSRS. The remaining 81 patients (35.5%) were treated with a fractionated technique. Protocols to treat meningioma were classified as 12–16 Gy per fraction for SSRS and 7 Gy/fraction/day for three consecutive days to reach a total dose of 21 Gy for FSRT. In univariate and multivariate analyses, tumor volume was found to be associated with local control rate (hazard ratio = 4.98, p = 0.025). The difference in actuarial local control rate (LCR) between the SSRS and FSRT groups after propensity score matching (PSM) was not statistically significant during the 2-year (96.86% versus 100.00%, respectively; p = 0.175), 5-year (94.76% versus 97.56%, respectively; p = 0.373), and 10-year (74.40% versus 91.46%, respectively; p = 0.204) follow-up period. FSRT and SSRS were equally well-tolerated and effective for the treatment of intracranial benign meningioma during the10-year follow-up period.

## Introduction

A meningioma is the most common benign brain tumor, accounting for 12–20% of all intracranial neoplasms^1,2^. The majority of meningiomas are benign, histologically classified as World Health Organization grade I and slow growing^3^. Current guidelines show that the gold standard procedure for the treatment of large (diameter > 3 cm) or symptomatic meningioma is gross total resection (GTR)^4,5^. However, complete resection may be limited by the size and site of the tumors. According to a previous study, Simpson grade 1 resection can be achieved in < 50% of the patients^6^ with local recurrence rates after GTR (Simpson grade I–III) ranging from 7–23% at 5 years, 20–39% at 10 years, and 24–60% at 15 years. On the other hand, local progression rates following subtotal resection (STR, Simpson grade IV–V) vary from 37–62% at 5 years, 52–100% at 10 years, and > 70% at 15 years^7^.

Given the relatively poor outcome of subtotal resection, radiation therapy, primarily fractionated stereotactic radiotherapy or single-session stereotactic radiosurgery (SSRS), has been shown to provide satisfactory outcomes when GTR is not feasible. However, there are limitations of radiation therapy, including risk of radiation injury, second brain tumor and difficulty in treating specific groups of lesions with a larger volume or a critical location^8^. These challenges are addressed by a relatively novel procedure, fractionated stereotactic radiotherapy (FSRT), which combines the precision of stereotactic positioning with the effect of fractionation to achieve steep dose gradients of arc-based radiation.

There is no clear consensus on the indications or effectiveness of FSRT or SSRS. Therefore, in this study, we compared the long-term efficacy and safety of FSRT and SSRS in the treatment of grade I meningioma.

## Methods

### Patient population

This study was exempt from approval requirements by the Institutional Review Board and without permission of patients consent, given that it was an epidemiology study with no definable patient information. We included patients who underwent linear accelerator (LINAC)-based radiosurgery of FSRT and SSRS with the Novalis system at our institute between March 2006 and April 2017. Inclusion criteria were the development of fresh, post-operative residual, and recurrent, benign meningiomas after previous Simpson grade I/II/III resection. Exclusion criteria were the presence of atypical and anaplastic meningiomas treated with previous radiotherapy. We extracted 228 patients with a total of 245 tumors; of these 70 (28.6%) patients had newly diagnosed tumors, 115 (46.9%) had residual tumors after partial resection, and 60 (24.5%) had recurrent tumors after previous total resection. The clinical characteristics of the patients included in this study are summarized in Table 1. Meningiomas were confirmed after initial evaluation with computed tomography (CT) or magnetic resonance imaging (MRI).

**Table 1:** Patient and tumor characteristics.

Characteristics	N = 228	Characteristics	N = 245
**Patient**		**Tumor**	
Median F/U, month (range)	56 (1–149)	TV, median (range)	4.47 (0.27–58.23)
Age, mean (SD)	56.74 (13.325)	TV > 10 cm^3^	50 (20.4%)
< 65 years	164 (30%)	Type	
≤ 65 years	64 (11.7%)	Primary	70 (28.6%)
Gender		Residual	115 (46.9%)
Female	158 (69.30%)	Recurrent	60 (24.5%)
Male	70 (30.70%)	Side	
Fractions		Midline	21 (8.6%)
1	147 (64.5%)	Right	112 (45.7%)
3	81 (35.5%)	Left	112 (45.7%)

*F/U *follow-up, *SD *standard deviation, *TV *tumor volume.

All methods were carried out in accordance with the European Association of Neuro-Oncology (EANO) guidelines recommending treatment of smaller meningiomas with SSRS^9^. Protocols to treat meningioma were classified as 12–16 Gy/fraction for a single day for SSRS and 7 Gy/fraction/day for 3 consecutive days to reach a total dose of 21 Gy for FSRT. Patients underwent FSRT if the diameter of the meningioma was > 3 cm, minimum lesion optic distance (MLOD) was ≤ 3 mm, and if the patients had lesions that compressed the brainstem.

### Radiosurgery technique

Thin-sliced CT and high-resolution MRI imaging with 1.0 mm in slice thickness and gadolinium contrast were employed for contouring the gross target volume (GTV). CT to MRI fusion was used to eliminate the potential distortion of MRI images. GTV was calculated using the iPlan Radiotherapy Planning Software (iPlan RT Image 3.0.1, Brainlab, Germany). The Novalis treatment system (Brainlab, Heimstetten, Germany) along with the software Brainscan, version 5.31 (Brainlab, Germany) was used to design the treatment plan. The use of precise micro-multileaf collimators in the Novalis system is able to shape the dose and minimize exposure of surrounding normal tissues by a single isocenter. A 100% isodose line was employed to cover the target.

During treatment, the patients were immobilized with a Brainlab thermoplastic fixation mask and an oral bite. A stereoscopic X-ray system combined with an infrared position tracking system was used to ensure the correct position.

Oral dexamethasone (0.5 mg 4-times-a-day for 3 consecutive days in SSRS and 7 consecutive days in FSRT) was given to the patients from the first day of treatment to prevent acute toxic effects induced by radiosurgery.

### Study outcomes

**Figure 1:**
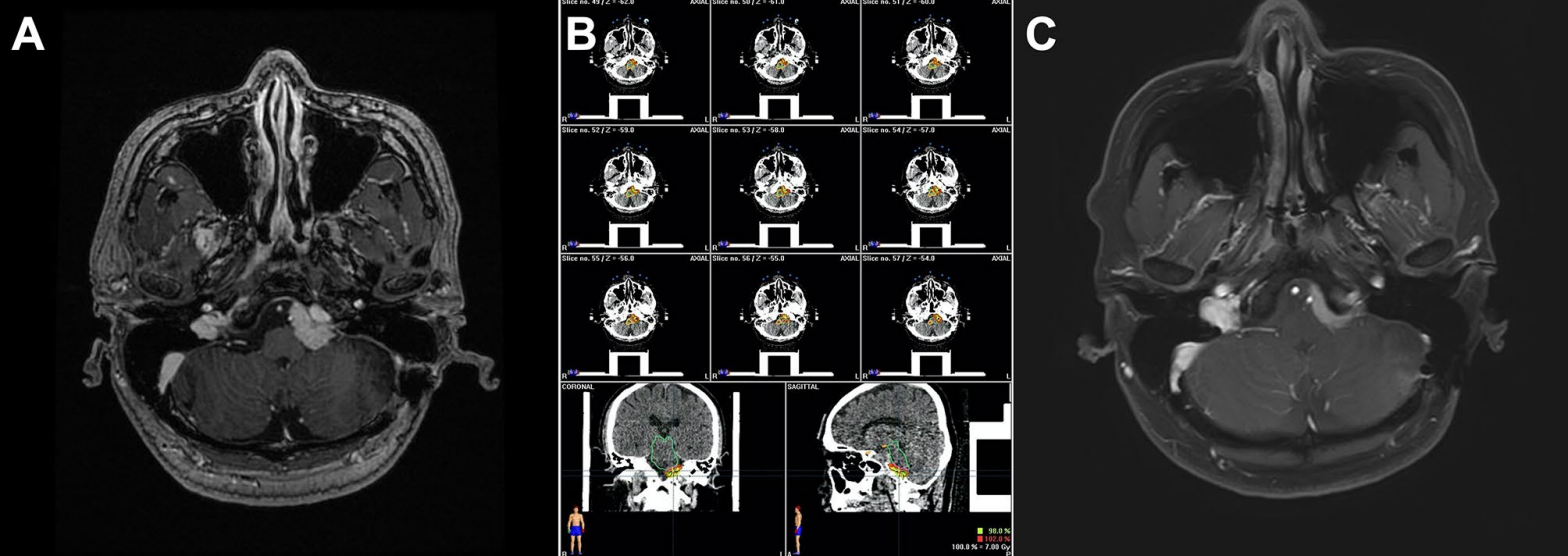
A 48-year-old male with a petroclival meningioma (volume, 3.02 ml). Treatment was 21 Gy in three fractions. (**A**) Pre-treatment magnetic resonance imaging (MRI) scan. (**B**) Treatment plan showing the resulting isodose line. (**B**) MRI scan 36-months after treatment showing tumor regression.

**Figure 2:**
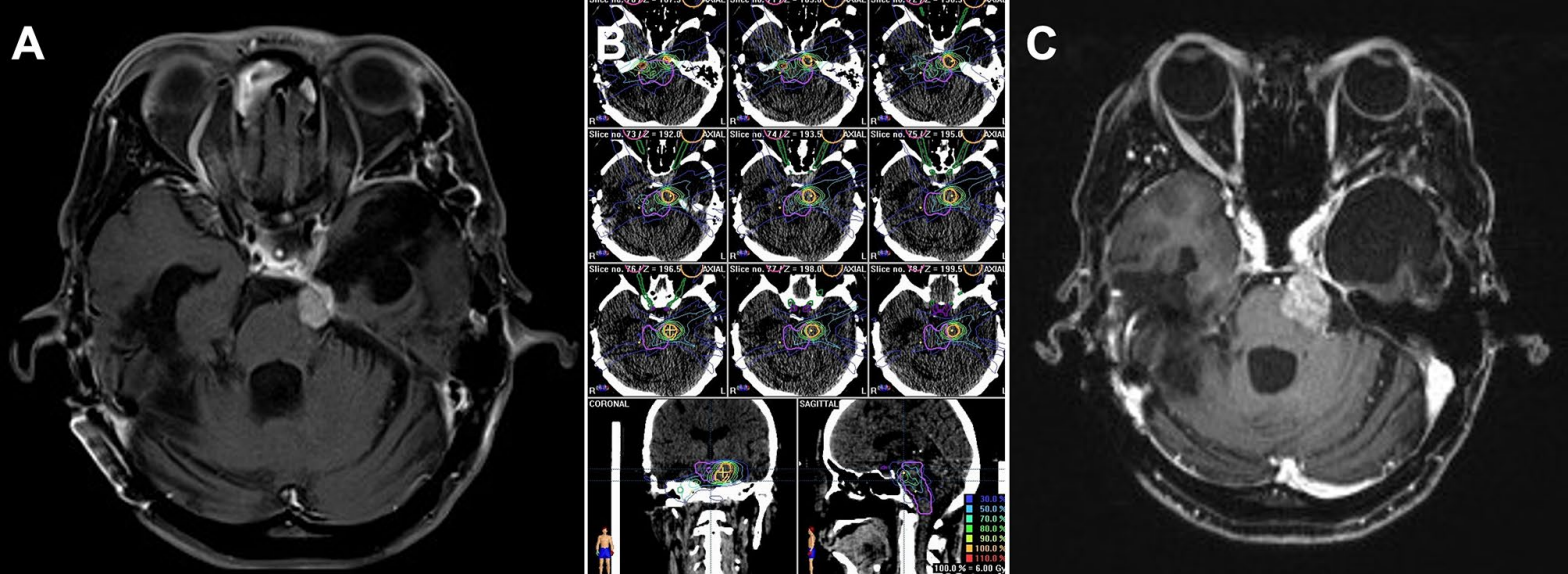
A 47-year-old female with a cerebellopontine angle meningioma (volume, 2.69 ml). Treatment was 21 Gy in three fractions. (**A**) Pre-treatment magnetic resonance imaging (MRI) scan. (**B**) Treatment plan showing the resulting isodose line. (**B**) MRI scan 98-months after treatment showing tumor progression.

Study outcomes included analyzing tumor progression, local control rate (LCR), and radiation-related complications. After SSRS/FSRT, MRI was used performed annually to evaluate tumor volume and obliteration of the meningiomas (Figs. 1, 2). The latest MRI images were used to analyze the remaining tumor volume. Obliteration was confirmed if no tumor progression was detected in the follow-up MRI. Follow-up duration was defined as the time from SSRS or FSRT to the last outpatient clinic date or the date when the last MRI was performed.

Progression was defined as volume progression ≥ 20% with or without clinical symptoms^10^. For patients with tumor progression, LCR was calculated based on the number of days between the date of SSRS or FSRT and the date of the latest MRI where progression was noted. For patients free of tumor progression, LCR was calculated based on the number of days between the date of SSRS or FSRT and the date of the latest outpatient department follow-up.

Onset of neurological deficits; clinical symptoms; imaging findings, including hemorrhage, peritumoral edema (PTE), and radionecrosis, cyst formation induced by radiation; and signal changes on T2-weighted images were recorded.

### Statistical analysis

**Figure 3:**
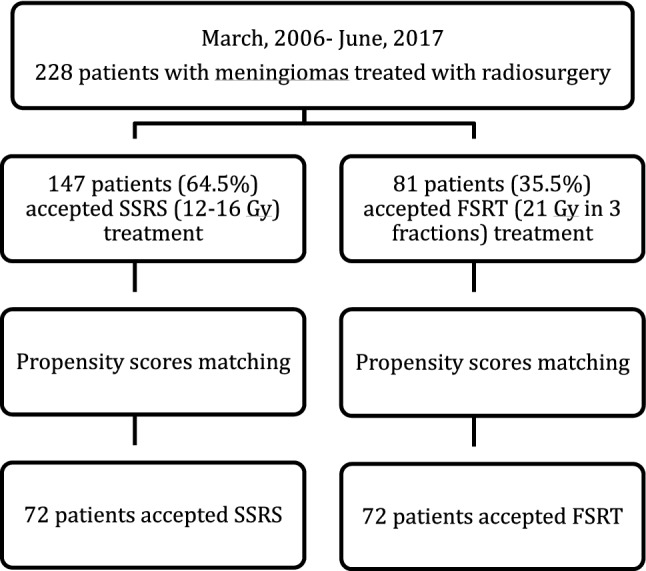
Flow chart demonstrating patients included in statistical analysis. *SSRS *single-session stereotactic radiosurgery, *FSRT *fractionated stereotactic radiotherapy..

To address confounding by indication and allocation bias in the treatment groups, we performed a propensity score matched (PSM) analysis^11^. Propensity scores were calculated by logistic regression analysis and included age, tumor volume, and tumor type (newly diagnosed, residual tumors after partial resection or recurrent tumors after total resection). Using the 1:1 nearest matching algorithm, each patient treated with SSRS was matched with patient treated with FSRT (Fig. 3). The SSRS and FSRT groups were compared using the Fisher’s exact test and Wilcoxon signed-rank test for categorical and continuous variables, respectively. The Kaplan–Meier method was used to analyze actuarial LCR for both groups. Statistical analyses were performed using SPSS software (IBM Corp. Released 2017. IBM SPSS Statistics for Windows, Version 25.0. Armonk, NY: IBM Corp). Two-sided *p* value < 0.05 was considered significant.

### Ethical approval

This study was approved by the Institutional Review Board of Chang Gung Memorial Hospital (IRB No. 201700082B0 obtained on Feb 8th, 2017). For this type of study formal consent is not required.

## Results

Overall, 228 patients with 245 tumors were included in this study; 147 (64.5%) and 81 (35.5%) patients received SSRS and FSRT, respectively. The mean age of the patients was 56.7 years and the median follow-up period of 56 months. The patients were mostly female 158 (69.3%). A total of 175 tumors (71.4%) were resected previously, while the primary treatment of the remaining 70 tumors (28.6%) was SSRS or FSRT. The location of the meningiomas (n = 245) are shown in Table 2.

**Table 2:** Anatomical distribution of 245 meningiomas treated with SSRS and FSRT.

Tumor location	Overall N (%)	SSRS N (%)	FSRS N (%)
Convexity	32 (13.1)	29 (18.1)	3 (3.5)
Falx	18 (7.3)	17 (10.6)	1 (1.2)
Parasagittal	37 (15.1)	33 (20.6)	4 (4.7)
Skull base	154 (62.9)	77 (48.1)	77 (90.6)
Ventricle	3 (1.2)	3 (1.9)	0 (0)
Cerebellum	1 (0.4)	1 (0.6)	0 (0)
Total	245 (100)	160 (100)	85 (100)

*SSRS *single-session stereotactic radiosurgery, *FSRT *fractionated stereotactic radiotherapy.

From a clinical point of view, we reported the patients initial major symptoms and the evolution during and after FSRT in Table 3. We noted a general improvement of symptoms in 21.4% of cases and stability in 75% and worsening in 3.6%. Two patients with tumor progression displayed neurological deficits (visual function and epilepsy). None of patient who received FSRT developed PTE. After FSRT, patients reported a 2.5% complication rate, including neurological deficits and PTE.

**Table 3:** A Summary changes in neurological deficits after FSRT.

Deficit	Pre-FSRT	Improved	Unchanged	Worsened	New
Visual function	13	2	10	1	1
Ocular movement	7	0	7	0	0
Hearing disorders	3	0	3	0	0
Tinnitus	3	1	2	0	0
Trigeminal neuralgia	16	5	10	1	0
Facial palsy	5	1	4	0	0
Epilepsy	6	3	3	0	1
Motor disorder	3	0	3	0	0

*FSRT *fractionated stereotactic radiotherapy.

### Radiological response

Regarding post-treatment tumor condition, tumor regression was observed in 19 (8.3%) patients, stationary tumor in 187 (82.0%) patients, and tumor progression in 22 (9.6%) patients. In the SRS group, tumor regression was observed in 15 (10.2%) patients, stationary tumor in 113 (76.9%) patients, tumor progression in 19 (12.9%) patients. In the FSRT group, tumor regression was observed in four (4.9%) patients, stationary tumor in 74 (91.4%) patients, and tumor progression in three (3.7%) patients.

### Propensity score matching analysis

Before matching, there were significant differences in patient characteristic with respect to age and median tumor volume; the SSRS group being older (58.2 ± 12.8 versus 54.2 ± 13.9 years, respectively; p = 0.032) and with a smaller median tumor volume (3.57 versus 7.70 cm^3^, respectively; p < 0.001), and longer median follow-up period (60.00 versus 45.00 months, respectively; p = 0.049) than the FSRT group. There were no significant differences in gender, tumor type, side, and location (skull base/non-skull base).

**Figure 4:**
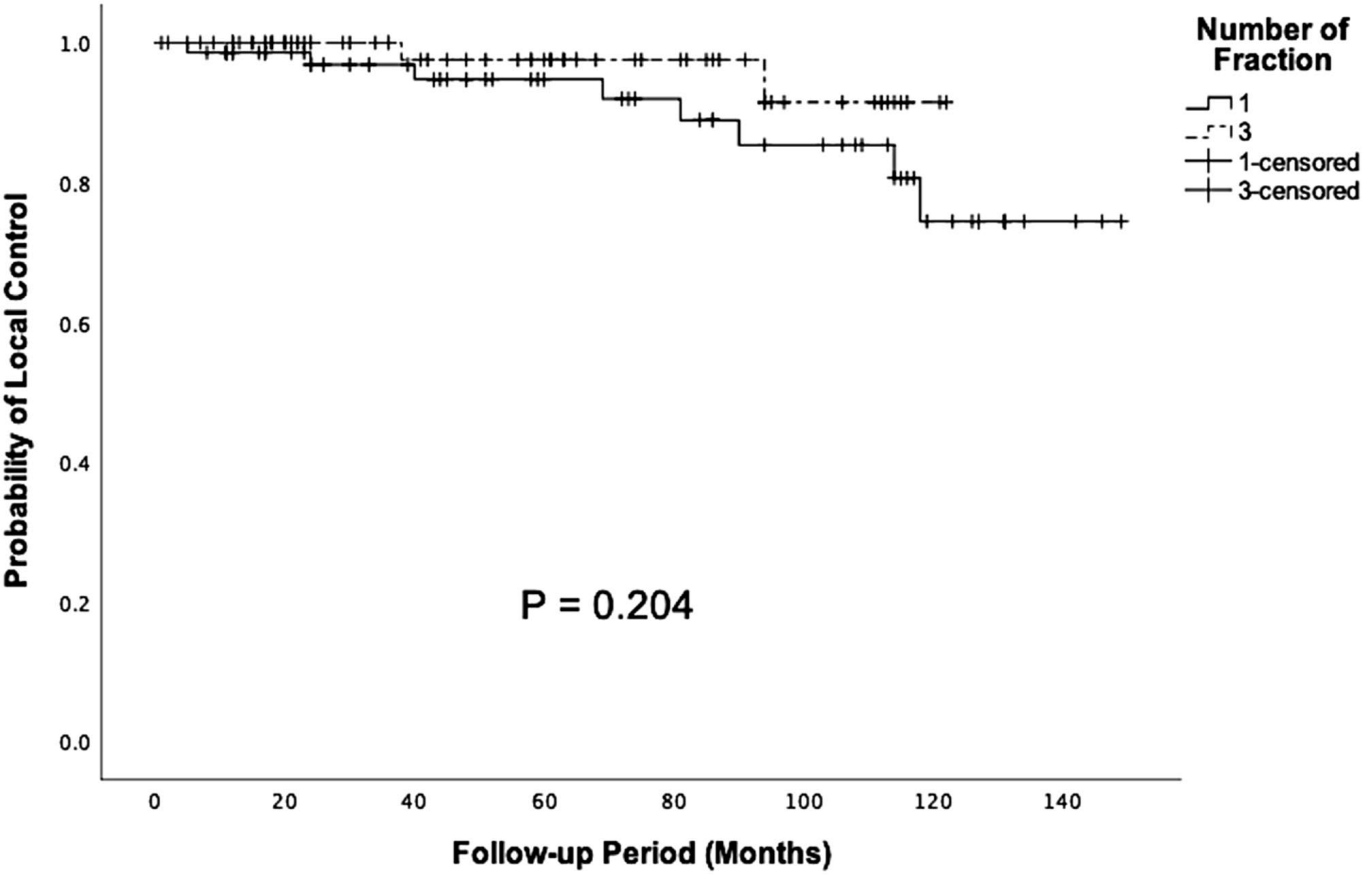
Kaplan–Meier analysis of 10-year actuarial LCR in SSRS and FSRT group after propensity-score matching (p = 0.204).

For PSM, 72 patients were selected from each group for further analysis (Table 4). After matching, there were no significant differences in age (p = 0.584), median tumor volume (p = 0.749), and median follow-up period (p = 0.768). The actuarial LCR of the SSRS group was 96.86%, 94.76%, and 74.40% in 2, 5, and 10 years, respectively. The actuarial LCR of the FSRT group was 100%, 97.56%, and 91.46% in 2, 5, and 10 years, respectively. When investigating patient outcome between the SSRS and FSRT groups after matching, there were no significant differences in 2 (p = 0.175), 5 (p = 0.373), and 10 years (p = 0.204) actuarial LCR (Fig. 4).

**Table 4:** Demographic characteristics of the SSRS and FSRT groups.

Characteristics	Before matching		p value	After matching		p value
	SSRS	FSRT		SSRS	FSRT	
No. of patients	147	81	N/A	72	72	N/A
Mean age, mean (SD)	58.15 (12.85)	54.19 (13.94)	0.032*	53.07 (12.05)	54.22 (13.14)	0.584
Male	41 (27.9)	29 (35.8)	0.215	23 (31.9)	23 (31.9)	1.000
**Type**			0.226			0.596
Newly diagnosed	48 (32.7)	18 (22.2)		13 (18.1)	18 (25.0)	
Residual	69 (46.9)	46 (56.8)		40 (55.6)	37 (51.4)	
Recurrent	30 (20.4)	17 (21.0)		19 (26.4)	17 (23.6)	
**Location**			0.472			0.247
Non-skull base	91 (61.9)	9 (11.1)		26 (36.1)	6 (8.3)	
Skull base	56 (38.1)	72 (88.9)		46 (63.9)	66 (91.7)	
TV (cm^3^), median (range)	3.57 (0.27–51.7)	7.70 (0.41–58.23)	< 0.001*	5.13 (0.27–51.7)	6.69 (0.41–49.55)	0.749
TV > 10 cm^3^	16 (37.2)	27 (62.8)	< 0.001*	N/A	N/A	N/A
F/U, month, median (range)	60.00 (1–149)	45.00 (2–125)	0.049*	58.50 (1–149)	49.50 (2–122)	0.768

*F/U *follow-up, *NA *not applicable, *TV *tumor volume.

### Predictors of tumor progression and pattern of local control

**Figure 5:**
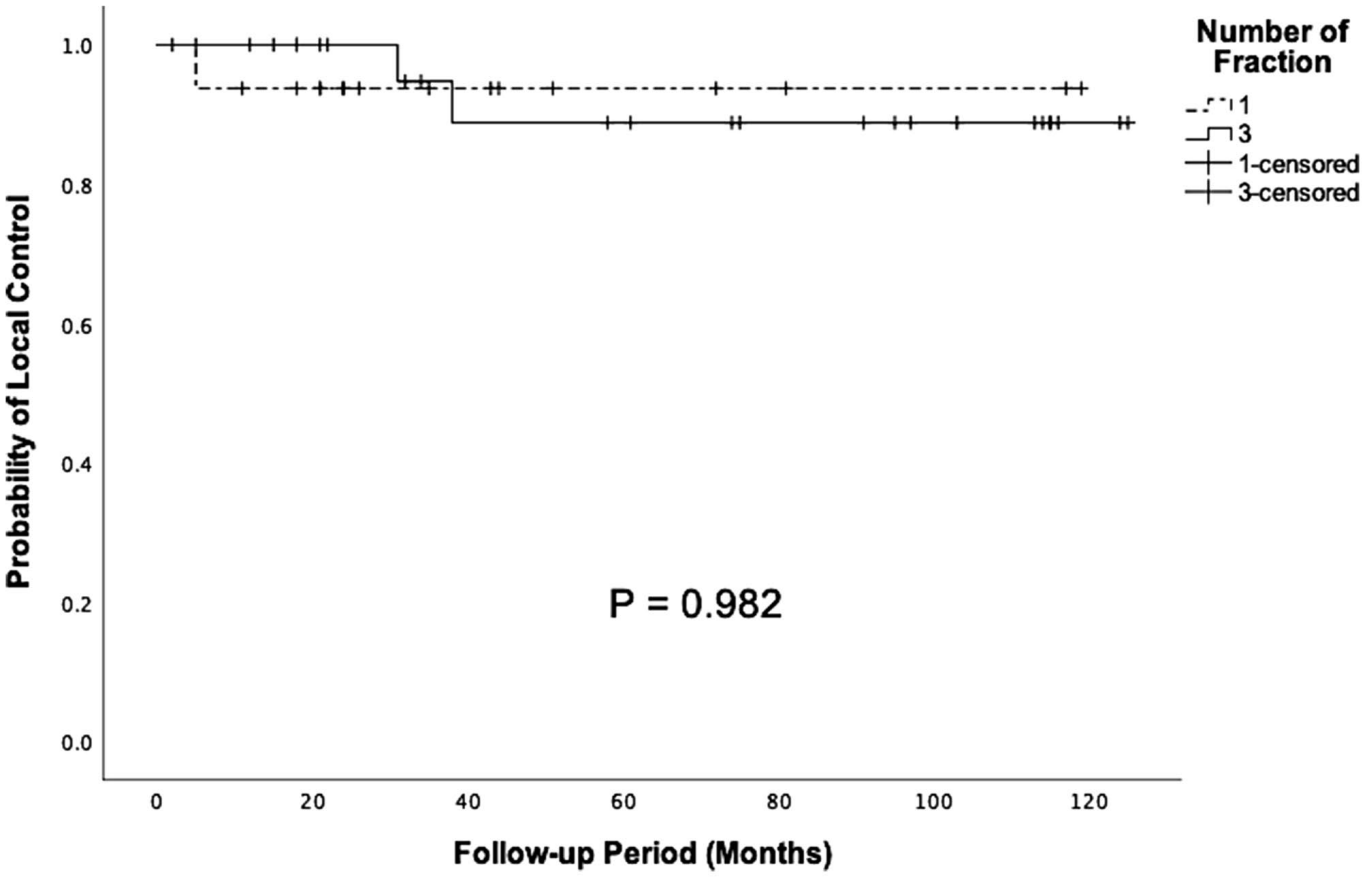
Kaplan–Meier analysis of clinical outcome in patients with large meningioma (tumor volume > 10 cm^3^) after SSRS and FSRT (p = 0.982).

Based on tumor size, in univariate analysis, LCR was found to be statistically significantly different. The 5-year actuarial LCR of tumors with volume > 10 cm^3^ was lower than the smaller tumors (98.2% versus 81.7%, p < 0.001). Age, gender, fractions, prior surgery, total marginal dosage, and location did not significantly influence treatment outcome in our analysis. In multivariate analysis, tumor size > 10 cm^3^ was found to be the only predicting factor for poor local control rates (hazard ratio = 4.98, 95% Cl = 1.25–20.7, p = 0.025). Other predictive variables related to patient outcomes were not statistically significant (Table 5). There was no significant difference in actuarial LCR in patients with tumor volume > 10 cm^3^ who received SSRS or FSRT (10-year actuarial LCR = 93.8% versus 88.8%, respectively; p = 0.982; Fig. 5). Similarly, there were no significant differences in actuarial LCR in patients with tumor volume < 10 cm^3^ who received SSRS or FSRT (10-year actuarial LCR = 91.8% versus 88.9%, respectively; p = 0.931).

**Table 5:** Predictors of unfavorable outcome.

Factors	Univariate	Multivariate		
	p value	HR	95% Cl	p value
Age > 65 years	0.293	2.003	0.264–17.38	0.519
Female	0.977	0.856	0.213–3.444	0.826
Tumor volume > 10 cm^3^	< 0.001*	4.982	1.253–20.70	0.025*
Fractions 1 versus 3	0.063	1.430	0.438–4.673	0.554
Prior surgery	0.251	2.242	0.275–18.30	0.451
Total marginal dose ≥ 14 Gy	0.462	1.600	0.157–16.30	0.691
Location^†^	0.781	1.148	0.440–2.997	0.778

^†^Location group as skull base/non-skull base.

## Discussion

In this study, the difference in tumor volume in patients who received SSRS and FSRT may limit the direct comparison of their outcomes. As tumor size is the only prognostic factor in our study, we performed a PSM in order to account for these confounding factors and to better ascertain the differences in LCR between treatment with SSRS and FSRT. After matching, we observed that actuarial LCR between the SSRS group and FSRT group did not significantly differ during the 2-year, 5-year, and 10-year follow-up period. Therefore, it appears that using 21 Gy divided to three consecutive fractions per the FSRT technique could provide similar effective results as SSRS up to 10 years of follow up. To the best of our knowledge, there was no previous study about propensity-matched outcome analysis assessing FSRT compared with SSRS.

### Fractionated stereotactic radiosurgery

The novel technique of FSRT is used for the treatment of large-size lesions or tumors proximity to eloquent structures^12^. We always tried to apply fractionated techniques to lower PTE rates while maintaining LCR^13–15^. Columbo et al.^16^ conducted a study where 49 patients were treated with SSRS (11–13 Gy) and 150 patients were treated with FSRT (14–25 Gy in 2–5 fractions); the 5-year actuarial LCR of patients in both groups was 93.5% with very few treatment-related complication (0.5% complication rate). Morimoto and colleagues published a retrospective series evaluating 32 benign meningiomas treated with FSRT (21–36 Gy in 3–5 fractions)^17^. After a 5-year follow-up period, tumor control rate was 87%. Since fractionated schemes were found to provide effective LCR in previous studies, FSRT has been described as yet another potentially useful tool.

On the basis of our previous experience, 14 Gy could provide a satisfactory outcome during SSRS. While considering the choice of radiation dose for FSRT, the α/β ratio is an important determinant. Thus, we chose the dose-fraction formula in accordance with the linear quadratic formalism presented previously^18^. Assuming an α/β ratio of 3.0, 21 Gy in three fractions is equivalent to 13.1 Gy in one fraction. Prescription dose and fraction numbers have been shown to vary across patients in previous studies; however, in our study we used three fractions consistently in the FSRT group^13,14,19^. Comparable actuarial LCR with the extended follow-up period was reported in our study. Using the Kaplan–Meier curve, we noted that LCR of FSRT was only slightly higher than SSRS during the 10-year follow-up period, while a significant disease progression was observed after 10 years. A longer follow-up period is required due to the slow growing nature of meningiomas.

### Local control of large meningiomas

**Figure 6:**
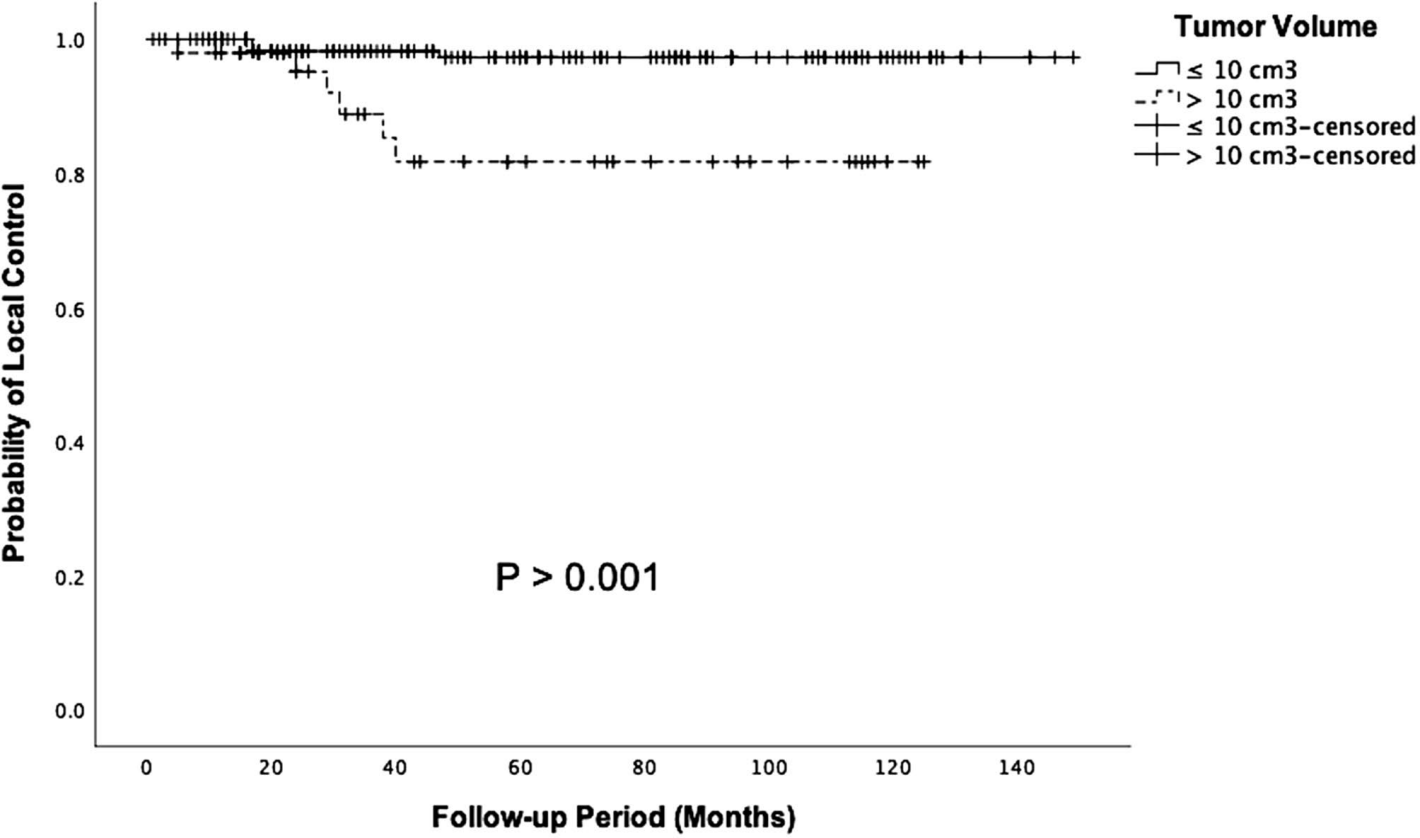
Kaplan–Meier analysis of clinical outcome in patients with tumor volume > 10 cm^3^ or tumor volume ≤ 10 cm^3^ (p < 0.001).

Target volume has been reported as the most important risk factor for treatment failure. DiBiase et al.^20^ reported that better 5-year disease free survival is observed in the smaller meningioma group (< 10 cm^3^) compared with the larger meningioma group (> 10 cm^3^) (91.9% versus 68%, respectively; p = 0.038). Similarly, in our study, we showed that patients with tumor volume $\le \hspace{0.17em}$10 cm^3^ experienced a significantly better 5-year actuarial LCR than those with larger tumors (Fig. 6).

There are relative few studies on the LCR of large benign meningiomas treated with FSRT compared with SSRS. Manabe et al. using LINAC-based FSRT (25 Gy in 2–10 fractions) in 14 patients with tumor volume > 13.5 cm^3^ reported that the 3-year progression-free survival rate was 69%^21^. In a systemic review, despite the potential systemic error in the measurement, Fatima et al. proposed that the larger tumors treated with FSRT (5–10 fractions) are significantly associated with better LCR and with higher extent of tumor shrinkage compared with the smaller tumors treated with SRS (< 5 fractions; p = 0.007 and p = 0.72, respectively)^22^. Han et al. compared the effectiveness of SSRS (n = 42) and FSRT (n = 28) in the treatment of large meningiomas (> 10 cm^3^); there were 16 skull base meningioma in FSRT group. The FSRT group had a higher 5-year actuarial LCR (92.9% versus 88.1%, respectively; p = 0.389)^23^. Conversely, we showed that the outcomes of larger meningiomas (> 10 cm^3^) prior to matching were worse in the FSRT group compared with the SSRS group. The difference in outcomes could be explained by the difference in mean tumor volume. In the study by Han et al., the mean tumor volume of patients treated with SSRS and FSRT was 15.2 cm^3^ and 21 cm^3^, respectively; whereas, in our study the mean tumor volume of patients treated with SSRS and FSRT was 21 cm^3^ and 19 cm^3^ respectively. Further prospective studies with large sample size and a long-term follow-up period are required to clarify the efficacy of SSRS and FSRT in the tumor control.

### Radiation associated peritumoral edema

FSRT is widely used in clinical practice; previous studies reported that FSRT has a lower rate of PTE than SSRS^17,24^. In a study with 173 patients, Unger et al.^13^ noted that PTE was significantly less common following FSRT (25 Gy in 5 fractions) than SSRS (median 15 Gy). This is in accordance with our results; none of the patients who underwent FSRT experienced edema, while two patients developed PTE in the SSRS group.

A single doses > 14 Gy has been recognized as a predictor for radiation-induced brain edema^24^. Other doses demonstrated no correlation between prescribed dose and PTE^4,25^. The two patients with PTE in our study received radiation dosage > 14 Gy (Table 6). Besides, both tumors were located in parasagittal; previous studies showed that parasagittal meningiomas is also associated with an increased higher risk of PTE, occurring in 16–50% of these patients^4,25^. The FSRT group may have a lower incidence of PTE compared with the SSRS group, though the result did not reach statistical significance probably due to the small sample size.

**Table 6:** Complications in two patients undergoing SSRS for benign meningioma.

	Age	Gender	Location	SRS dose (Gy)	Type	Tumor volume (cm^3^)	Outcome
Patient 1	45	Female	Parasagittal	15	New diagnosis	0.635	PTE
Patient 2	69	Female	Parasagittal	16	Recurrent	3.022	PTE

*PTE *peritumoral edema.

## Limitations

**Figure 7:**
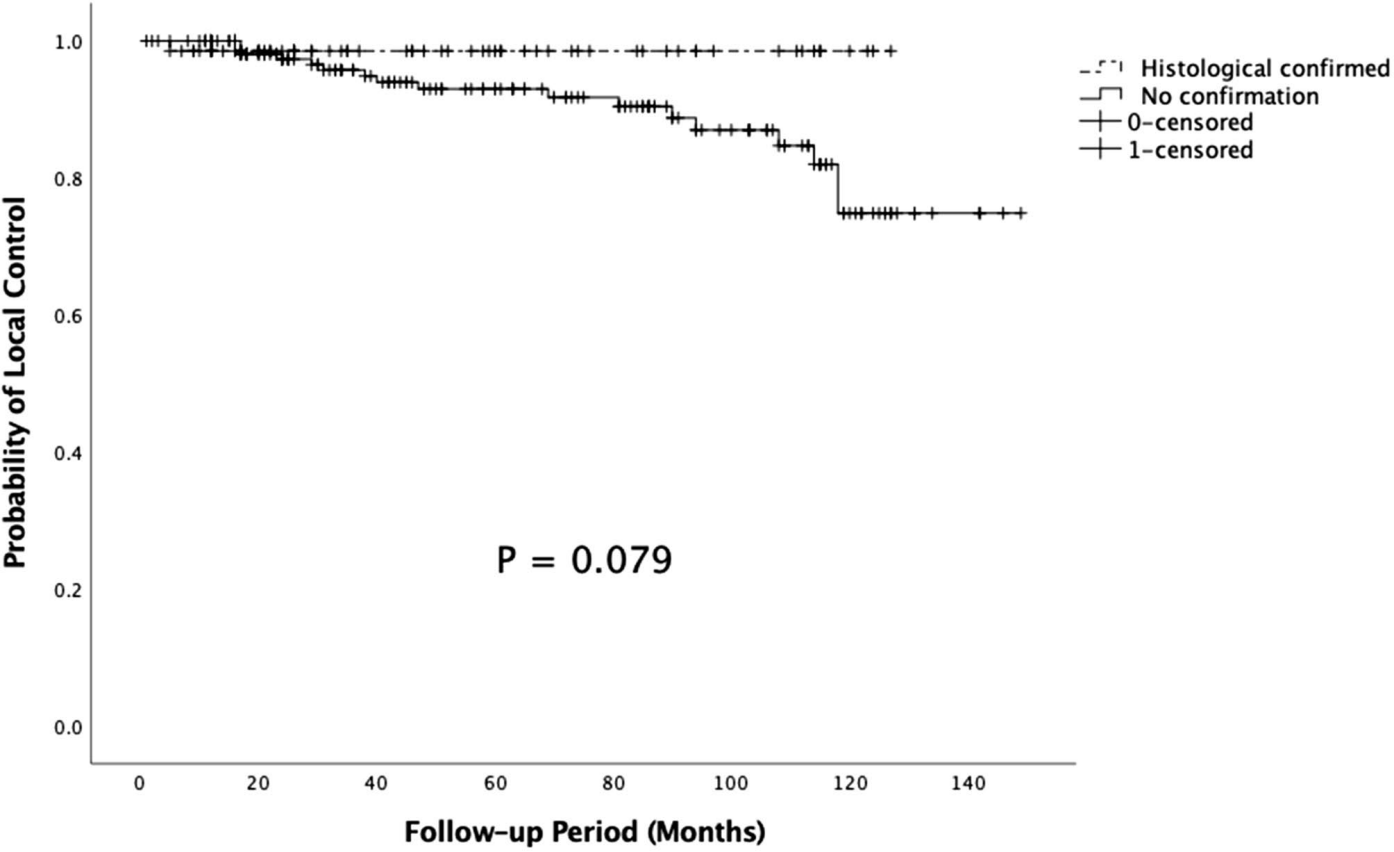
Kaplan–Meier analysis of 10-year actuarial LCR in patients with histological confirmation and non-confirmation in FSRT group (p = 0.079).

The major limitation of this study is its retrospective design due to which selection bias might exist. Not all the tumors detected by imaging were confirmed histologically to be benign meningiomas prior to treatment. Overall control rate was analyzed for those patients with histological confirmation of the diagnosis and those without confirmation. In the entire cohort, patients without confirmation showed a trend toward decreased actuarial LCR compared with patients with histological confirmation. However, there was no significant difference between the two groups in five (p = 0.251) and 10 years (p = 0.079) actuarial LCR (Fig. 7). Imbalance proportion of skull base between SSRS and FSRT group was also another bias but it was insignificant in our unfavorable outcome factors analysis. Moreover, given that grade I meningiomas are slow progressing tumors, the relatively shorter follow-up period can be a limitation of this study. Although we have attempted to control the differences in patient characteristics that could potentially influence the rates of PTE, no significant correlation was found between fractions and PTE; this may be due to the sample size of the study. Further studies with longer follow-up period and larger sample size are needed for further evaluation of tumor.

## Conclusions

Use of FSRT (21 Gy for 3 consecutive days) for the treatment of large or critically located benign meningiomas could provide effective and well-tolerated outcomes similar to SSRS. FSRT had fewer but not significant side effects and a lower incidence of PTE. Larger meningiomas treated with FSRT were generally well tolerated. Prospective, randomized studies with longer follow-up period and larger sample size are needed for the further evaluation.

## Abbreviations

CT: Computed tomography
FSRT: Fractionated stereotactic radiotherapy
GTR: Gross total resection
LCR: Local control rate
LINAC: Linear accelerator
MLOD: Minimum lesion optic distance
MRI: Magnetic resonance imaging
PSM: Propensity Score Matching
PTE: Peritumoral edema
SRS: Stereotactic radiosurgery
STR: Subtotal resection
TV: Tumor volume
WHO: World Health Organization
